# ^177^Lu-labeled PSMA targeting therapeutic with optimized linker for treatment of disseminated prostate cancer; evaluation of biodistribution and dosimetry

**DOI:** 10.3389/fonc.2023.1221103

**Published:** 2023-09-27

**Authors:** Ayman Abouzayed, Kamila Seitova, Fanny Lundmark, Vitalina Bodenko, Maryam Oroujeni, Vladimir Tolmachev, Ulrika Rosenström, Anna Orlova

**Affiliations:** ^1^ Department of Medicinal Chemistry, Uppsala University, Uppsala, Sweden; ^2^ Scientific and Research Laboratory of Chemical and Pharmaceutical Research, Siberian State Medical University, Tomsk, Russia; ^3^ Research Centrum for Oncotheranostics, Research School of Chemistry and Applied Biomedical Sciences, Tomsk Polytechnic University, Tomsk, Russia; ^4^ Department of Immunology, Genetics and Pathology, Uppsala University, Uppsala, Sweden; ^5^ Affibody AB, Solna, Sweden; ^6^ Science for Life Laboratory, Uppsala University, Uppsala, Sweden

**Keywords:** prostate cancer, PSMA, lutetium-177, PC3-pip cells, dosimetry

## Abstract

**Introduction:**

Prostate specific membrane antigen (PSMA), highly expressed in metastatic castration-resistant prostate cancer (mCRPC), is an established therapeutic target. Theranostic PSMA-targeting agents are widely used in patient management and has shown improved outcomes for mCRPC patients. Earlier, we optimized a urea-based probe for radionuclide visualization of PSMA-expression *in vivo* using computer modeling. With the purpose to develop a targeting agent equally suitable for radionuclide imaging and therapy, the agent containing DOTA chelator was designed (BQ7876). The aim of the study was to test the hypothesis that ^177^Lu-labeled BQ7876 possesses target binding and biodistribution properties potentially enabling its use for radiotherapy.

**Methods:**

BQ7876 was synthesized and labeled with Lu-177. Specificity and affinity of [^177^Lu]Lu-BQ7876 to PSMA-expressing PC3-pip cells was evaluated and its processing after binding to cells was studied. Animal studies in mice were performed to assess its biodistribution *in vivo*, target specificity and dosimetry. [^177^Lu]Lu-PSMA-617 was simultaneously evaluated for comparison.

**Results:**

BQ7876 was labeled with Lu-177 with radiochemical yield >99%. Its binding to PSMA was specific *in vitro* and *in vivo* when tested in antigen saturation conditions as well as in PSMA-negative PC-3 tumors. The binding of [^177^Lu]Lu-BQ7876 to living cells was characterized by rapid association, while the dissociation included a rapid and a slow phase with affinities K_D1_ = 3.8 nM and K_D2_ = 25 nM. The half-maximal inhibitory concentration for ^nat^Lu-BQ7876 was 59 nM that is equal to 61 nM for ^nat^Lu-PSMA-617. Cellular processing of [^177^Lu]Lu-BQ7876 was accompanied by slow internalization. [^177^Lu]Lu-BQ7876 was cleared from blood and normal tissues rapidly. Initial elevated uptake in kidneys decreased rapidly, and by 3 h post injection, the renal uptake (13 ± 3%ID/g) did not differ significantly from tumor uptake (9 ± 3%ID/g). Tumor uptake was stable between 1 and 3 h followed by a slow decline. The highest absorbed dose was in kidneys, followed by organs and tissues in abdomen.

**Discussion:**

Biodistribution studies in mice demonstrated that targeting properties of [^177^Lu]Lu-BQ7876 are not inferior to properties of [^177^Lu]Lu-PSMA-617, but do not offer any decisive advantages.

## 1 Introduction

Prostate cancer (PCa) is a severe public health problem; approximately 500.000 men are diagnosed with this disease annually (1). Despite the fact of the high level of 5-year survival for local and regional PCa (99-100%), metastasized PCa has shown as low 5-year survival as 30% (2). The most common methods used for PCa treatment include radical prostatectomy and external beam radiation therapy for local disease, while hormone therapy, androgen deprivation therapy and chemotherapy are used for metastasized PCa. Current medical guidelines recommend antimitotic chemotherapy with docetaxel (3). However, the overall benefit for the patient is typically poor. In most cases, development of tumor resistance causes tumor progression. Due to low survival of metastatic PCa patients, an efficient treatment of metastatic PCa is one of unmet clinical needs of modern oncology (4).

Prostate specific membrane antigen (PSMA) is an established therapeutic target in PCa (5). PSMA (also known as glutamate carboxypeptidase II) is a type II transmembrane protein with an N-terminal cytoplasmic tail, a helical transmembrane structure, and an extracellular C-terminus (6). PSMA has two unique enzymatic functions, including NAALADase activity (cleaving terminal glutamate from the neurodipeptide, N-acetyl-aspartyl-glutamate, NAAG) and folate hydrolase activity, which results in cleavage of the terminal glutamates from g-linked polyglutamates (7). PSMA is located in the cytosol in normal prostate cells but is expressed as a membrane-bound protein in PCa (8). Normally, PSMA is expressed in the prostate (secretory-acinar epithelium), kidneys (proximal tubules), nervous system glia (astrocytes and Schwann cells) and the small bowel (jejunal brush border). Its function is only well defined in the nervous system and small bowel. The reason for PSMA expression in prostate epithelium and renal proximal tubules is not determined but may be connected with folate metabolism in these tissues, i.e. potential folate reuptake in the kidneys and release of monoglutamated folates into the seminal fluid (9).

PSMA is highly expressed in hormone-resistant and metastatic PCa (10). The level of PSMA expression rises with increasing tumor dedifferentiation in metastatic and hormone-refractory cancers (11). Increased PSMA expression in prostate cancer is associated with a higher tumor grade and a high risk of disease progression as defined by biochemical recurrence after radical prostatectomy (12). Different other types of cancers also show high expression of PSMA, i.e. follicular lymphoma, multiple myeloma, papillary and follicular thyroid carcinoma, pancreatic neuroendocrine tumor, gastrointestinal stromal tumor, and squamous cell carcinoma of the oropharynx (13).

Several small molecule inhibitors of PSMA have been developed and tested in clinical studies (13). Based on the zinc-binding moieties targeting the active site of PSMA, there are three types of small molecule inhibitors: phosphorous-based, thiol-based, and urea-based. The phosphorous-based type of inhibitors were discovered first and it was found that the phosphonate core was favorable for interacting with the two zinc ions in the active domain of PSMA. The discovery of the phosphorous-based inhibitors was followed by the development of thiol-based inhibitors to reduce the high polarity of the first type. Further attempts to identify alternative zinc-binding groups led to the introduction of urea-based PSMA inhibitors first reported by the Kozikowski’s group (14). Currently, urea-based PSMA-targeting agents for the diagnosis and therapy of PCa are widely used in patient management (15, 16).

The use of PSMA-targeted therapy using radioligands, such as [^177^Lu]Lu-PSMA-617, improved outcomes for patients with metastatic castration-resistant PCa (17, 18). Although patients with low PSMA expression are not eligible for this therapy, some patients do not respond to it and some develop resistance despite initially good response (19), [^177^Lu]Lu-PSMA-617 has demonstrated impressive results in the phase III VISION trial and has been approved by Food and Drug Administration for clinical use (20).

Earlier, we performed an optimization of the linker connecting the PSMA-binding moiety to the chelator necessary for labeling to improve the targeting features of urea-based probes for radionuclide visualization of PSMA expression *in vivo* (21). For that optimization, the macrocyclic triaza chelator NOTA was selected for the coordination of radionuclides. This chelator was selected because it permits efficient labeling with a number of positron-emitting radionuclides, such as ^68^Ga, ^18^F (using AlF chemistry), ^55^Co, ^64^Cu, as well as ^111^In for imaging using SPECT. Our findings have demonstrated that affinity to PSMA, biodistribution profile and tumor targeting efficiency are critically dependent on the linker composition. The best variant was designated as BQ7859. ^111^In-labeled BQ7859 accumulated in PCa xenografts in mice in a PSMA-dependent manner and provided a high contrast imaging of PSMA expression shortly after administration. It would therefore be attractive to use BQ7859 also for radionuclide therapy. However, the NOTA chelator is suboptimal for coupling of the most commonly used therapeutic radionuclide lutetium-177 since the Lu-NOTA complex is insufficiently stable (22). On the opposite, the macrocyclic tetra-aza chelator DOTA provides stable conjugation of lutetium-177 (22). In addition, DOTA is suitable for labeling with other therapeutic nuclides such as ^90^Y, ^225^Ac and ^227^Th. Moreover, DOTA enables sufficiently stable coupling of ^68^Ga, ^111^In and ^55^Co that could be useful for the development of imaging companion diagnostics.

**Figure 1:**
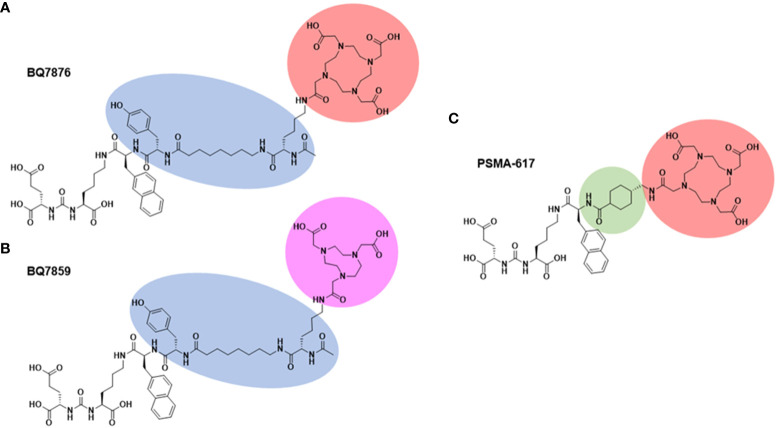
Chemical structure of BQ7876 **(A)**, BQ7859 **(B)**, and PSMA-617 **(C)**. Differences in the chemical compositions of the PSMA binders are highlighted.

With the aim to develop a targeting agent, which will be equally suitable for radionuclide therapy and radionuclide imaging, a new PSMA-targeting agent was designed and designated BQ7876 (**Figure 1**). This agent contains the same urea-based targeting moiety and linker system as BQ7859, but is equipped with the more versatile DOTA chelator. It has to be noted that the substitution of a radionuclide-chelator part in such small targeting agent could substantially affect the charge distribution and geometry of the probe (23). This might result in appreciable alteration of binding affinity to the molecular target, off-target interaction of the agent, and influence its biodistribution properties and tumor uptake. Thus, a full pre-clinical characterization of the novel tracer was required.

The aim of the study was to test the hypothesis that ^177^Lu-labeled BQ7876 possesses target binding and biodistribution properties which potentially could enable its use for radionuclide therapy. Specificity and affinity of [^177^Lu]Lu-BQ7876 binding to PSMA-expressing PC3-pip cells were evaluated and its processing after binding to the target was studied. Animal studies in mice were performed to assess the biodistribution *in vivo*, the specificity of its accumulation in PSMA-expressing tumor xenografts and dosimetry. As comparator, [^177^Lu]Lu-PSMA-617 was simultaneously evaluated.

## 2 Materials and methods

Lutetium [^177^Lu]LuCl_3_ and indium [^111^In]InCl_3_ were purchased from Perkin Elmer Sverige AB (Hägersten, Sweden). Data on radiochemical yield and purity were obtained using instant thin-layer chromatography (iTLC) on iTLC silica gel strips (Varian, Lake Forest, CA, USA). The distribution of activity along strips was measured using a Cyclone storage phosphor system (Packard Instrument Company, Downers Grove, IL, USA) and analyzed by OptiQuant image analysis software (Perkin Elmer, Waltham, MA, USA). Activity in organs and tissues was measured with the use of an automated gamma-spectrometer (2480 Wizard2, Wallac, Turku, Finland).

### 2.1 Targeting substance

Chemical structure of BQ7876 is shown in **Figure 1A**. The binding moiety and the linker of BQ7876 are the same as for BQ7859 (**Figure 1B**), which has been described by Lundmark and co-workers (21). Synthesis of BQ7876 was carried out by standard Fmoc solid-phase peptide synthesis (SPPS) using 2-chlorotrithyl resin (2-CTC) with a loading of 1.6 mmol/g as a solid support. BQ7876 was synthesized, purified and characterized in the same way as BQ7859 (21) except the conjugation of DOTA-bis(*t*-Bu)ester (CheMatech, Dijon, France) instead of NOTA-tri(*t*-Bu)ester. The product was purified using preparative reversed-phase high-performance liquid chromatography (RP-HPLC) (**Supplementary Figure 1**). Analytical liquid chromatography mass-spectrometry (LC-MS) (Impact II instrument, Bruker, Billerica, MA, USA) was used to confirm the identity and purity of the product. Analytical high-performance liquid chromatography (HPLC) was performed on a Dionex UltiMate 3000 HPLC system with a Bruker amazon SL ion trap mass spectrometer and detection by UV (diode array detector, 214, 254, and 280 nm) and electrospray ionization (ESI) MS using a Phenomenex Kinetex C18 column (50 × 3.0 mm, 2.6 µm particle size, 100 Å pore size) with gradients of H_2_O/CH_3_CN/0.05%HCOOH as mobile phase at a flow rate of 1.5 mL/min. Mass-spectrometry data (**Supplementary Figure 2**) confirmed the correct mass (calculated [M+H]^+^ and [M+2H]^2+^: 1378.56 and 689.78; observed [M+H]^+^ and [M+2H]^2+^: 1378.6 and 689.2), which demonstrated its authenticity. PSMA-617 (**Figure 1C**) was used as a comparator and was purchased from ABX advanced biochemical compounds GmbH (Radeberg, Germany).

### 2.2 Labeling

For labeling of BQ7876 and PSMA-617 with lutetium-177, the solution of [^177^Lu]LuCl_3_ (12 μL in 0.1 M HCl, 83–108 MBq) was mixed with a solution of BQ7876 or PSMA-617 (3 μL, 3 nmol) in 80 μL of 1M sodium ascorbate, pH 6.0, and incubated at 85°C for 30 min. The radiochemical yield and purity were determined using iTLC strips eluted with 0.2 M citric acid, pH 2.0. Radio-HPLC was performed using a Hitachi Chromaster HPLC system equipped with a Luna C18 column (5 µm, 100 Å, 150 x 4.6 mm; Phenomenex, Værløse, Denmark). The solvent gradients were 5–10% CH_3_CN (0–15 min), 70–95% 0.1% CH_3_CN (15–17 min), and 95%-0.1% CH_3_CN (19–20 min). The HPLC system was equipped with variable UV wavelength and radioactivity detectors.

For the determination of a half-maximal inhibitory concentration (IC_50_), PSMA-617 was labeled with indium-111. For this purpose, 2 nmol PSMA-617 were dissolved in 40 µL 0.2 M ammonium acetate, pH 5.5, and 12 µL (10 MBq) of [^111^In]InCl_3_ solution in 0.01 M HCl was added. The mixture was vortexed and incubated at 85 °C for 30 min. The yield was determined using iTLC eluted with 0.2 M citric acid, pH 2.0. Since the radiochemical yield was over 98%, [^111^In]In-PSMA-617 was used in further experiments without purification.

### 2.3 *In vitro* studies

The human prostate cancer cell line PC-3 was purchased from the American Type Culture Collection (ATCC; LGC Promochem, Borås, Sweden). The cells were cultured in Roswell Park Memorial Institute (RPMI) 1640 medium supplemented with 10% fetal bovine serum (FBS), 2 mM L-glutamine, penicillin 100 IU/mL and 100 µg/mL streptomycin at 37°C and 5% CO_2_ atmosphere. The PSMA-transfected and overexpressing variant of PC3 cell line, PC3-pip, was provided by Prof. M. Pomper, Department of Radiology, the Johns Hopkins University, Baltimore, MD, US. PC3-pip cells were cultured in two types of RPMI media. The first one contained 20% fetal bovine serum, 1% of L-glutamine (5 mL) and 1% of PEST (penicillin and streptomycin 5 mL). The second one contained 20% fetal bovine serum, 1% of L-glutamine (5 mL) and 1% of PEST (penicillin and streptomycin 5 mL) and 10 μg/mL of pyramycin. The second media is used every second passage to maintain clone selection. All *in vitro* experiments were performed in triplicates. For a typical experiment, cells were seeded one day before the experiment in Petri dishes (35 mm) in the amount of 1×10^6^ cell/dish.

#### 2.3.1 *In vitro* specificity test

*In vivo* specificity was tested using a saturation method. Before the experiment, cells were washed with PBS solution. A set of six cell dishes with PC3-pip cells was used for each substance. Cells in control dishes were pre-incubated with unlabeled compound, BQ7876 or PSMA-617 (100 nM) for 15 min to saturate PSMA. Thereafter, the radiolabeled substance ([^177^Lu]Lu-BQ7876, [^177^Lu]Lu-PSMA-617 or [^111^In]In-PSMA-617) was added to all cell dishes to a concentration of 1 nM. After one-hour incubation at 37°C cells were washed with PBS solution, detached and collected. Cell-associated activity was measured using gamma-counter and presented as the percentage of added activity.

#### 2.3.2 Determination of half-maximal inhibitory concentration (IC_50_)

For determination of IC_50_, BQ7876 and PSMA-617 were loaded with non-radioactive Lu of natural isotopic composition (^nat^Lu) by incubation with a three-fold molar excess of ^nat^LuCl_3_ at 85 °C for 30 min in 0.2 M ammonium acetate, pH 5.5. Cells were washed with a PBS solution and incubated at 4°C for 4 hours with [^111^In]In-PSMA-617 (1 nM) in the presence of increasing concentrations (range 0 to 2 μM) of competitors, ^nat^Lu-BQ7876 or ^nat^Lu-PSMA-617. Thereafter, cells were detached, collected, and cell-associated activity was measured using gamma-counter. The half-maximal inhibitory concentration was calculated using GraphPad software (version 9.3.1 for Windows, GraphPad Software, LLC. CA, USA).

#### 2.3.3 Binding affinity

Affinity of [^177^Lu]Lu-BQ7876 binding to PSMA was measured on PC3-pip cells at room temperature using LigandTracer White instrument (Ridgeview Diagnostics, Uppsala, Sweden). The affinity measurements were performed according to the methods described by Tolmachev and co-workers (24). After 20 min of background measurement, the first concentration of labeled compound was added. The measurements of association curves were done for three concentrations of [^177^Lu]Lu-BQ7876, 1, 3, and 10 nM, for 90 min each. Thereafter, media containing [^177^Lu]Lu-BQ7876 was removed, fresh media was added to the dish and dissociation was measured. The binding curve was analyzed and equilibrium dissociation constant (K_D_) was calculated using the TraceDrawer Software (Ridgeview Instruments, Vänge, Sweden). The interaction heterogeneity was estimated using Interaction Map analysis (Ridgeview Diagnostics, Uppsala, Sweden).

#### 2.3.4 Cellular processing

Internalization of radiolabeled PSMA binders was evaluated using an acid wash method (21). PC3-pip cells were incubated with 2 nM of [^177^Lu]Lu-BQ7876 or [^177^Lu]Lu-PSMA-617 in RPMI medium at 37°C (5% of CO_2_). At predetermined time points (1, 2, 6 and 24 h), cells were treated with 0.5 mL of 4 M urea solution in a 0.2 M glycine buffer, pH 2.5, for 5 minutes on ice. After that, the acidic supernatant was collected and this fraction was considered to be a membrane-bound activity. To lyse the cells, 0.5 mL of 1 M sodium hydroxide solution was added and incubated at 37°C for at least 20 min. Alkaline-containing solution was collected, and the basic fraction was considered to be an internalized activity. Samples were measured using gamma-counter.

### 2.4 *In vivo* studies

All animal experiments were planned and executed according to national legislation on laboratory animals’ protection. The studies were admitted by the Local Ethics Committee for Animal Research (Uppsala, Sweden) and followed the national legislation on protection of laboratory animals (protocol code 5.8.18-11931/2020, approved on 28 August 2020).

To prepare xenografts, PC3-pip and PC3 (PSMA-negative control) cells (10^7^ cells in 100 μL of media) were subcutaneously injected in the hind legs of female Balb/c nu/nu mice. At the time of the experiment, the average mouse weight was 18 ± 2 g. The average tumor weight was 0.2 ± 0.1 g.

#### 2.4.1 Biodistribution of [^177^Lu]Lu-BQ7876

Biodistribution of [^177^Lu]Lu-BQ7876 was determined at 1, 3, 24, 48 and 130 hours p.i. Mice were injected with [^177^Lu]Lu-BQ7876 (280 kBq/mouse). The amount of substance was adjusted to 80 pmol/mouse using unlabeled substance. A group of four animals per data point was used. The labeled peptide was injected into the tail vein. Mice were anaesthetized by a lethal intraperitoneal injection of ketamine and xylazine mixture solution (the dose of ketamine was 250 mg/kg and the dose of xylazine was 25 mg/kg) and sacrificed. Mice were exsanguinated by heart puncture using a heparinized syringe under anaesthesia. Organs and tissues were excised and weighed, and the activity of each organ was measured. The percentage of injected dose per gram of sample (%ID/g) was calculated. Activity in the rest of the gastrointestinal tract was measured to estimate hepatobiliary excretion. The rest of the body was also collected and its activity was measured.

For comparison, a group of mice with PC-3 xenografts was injected with [^177^Lu]Lu-PSMA-617 (280 kBq/mouse, 80 pmol/mouse) and the biodistribution was measured 3 h after injection.

#### 2.4.2 *In vivo* specificity test

To test if the accumulation of [^177^Lu]Lu-BQ7876 was PSMA-specific, the biodistribution in a group of mice bearing PSMA-negative PC3 xenografts was measured 3 h after injection using the same protocol.

#### 2.4.3 SPECT imaging

To confirm the biodistribution data, imaging of mice bearing PC3-pip xenografts was performed. The mice were injected with [^177^Lu]Lu-BQ7876 (900 kBq/mouse, 80 pmol/mouse) or [^177^Lu]Lu-PSMA-617 (1200 kBq/mouse, 80 pmol/mouse). Imaging was performed at 3 h pi using nanoScan SPECT/CT (Mediso Medical Imaging Systems, Hungary) according to the protocol describe earlier (25).

#### 2.4.4 Dosimetry estimation

Dosimetry of [^177^Lu]Lu-BQ7876 in humans was estimated as described earlier (25). Briefly, the data from mice were uncorrected for decay and up-scaled to humans by the “percent kg/g method” (26) according to following equation:


(%ID/organ)human = [(%ID/g)animal ×(kgTBweight)animal × (g organ/(kgTBweight)human]

The organ weight from reference adult male (ICRP publication 23) phantom was used for upscaling. The uptake value was fitted by an exponential function and areas under curves were calculated to determine residence times. OLINDA/EXM 1.0 software (Vanderbilt University, Nashville, TN, USA) was used to estimate absorbed doses.

### 2.5 Data analysis and statistics

Data were treated with the help of the impaired, two-tailed *t-*test using GraphPad Prism (version 8.00 for Windows GraphPad Software) to find significant statistical differences (p<0.05). Binding data were analyzed using the GraphPad Prism Software package.

## 3 Results

### 3.1 Radiolabeling

**Figure 2:**
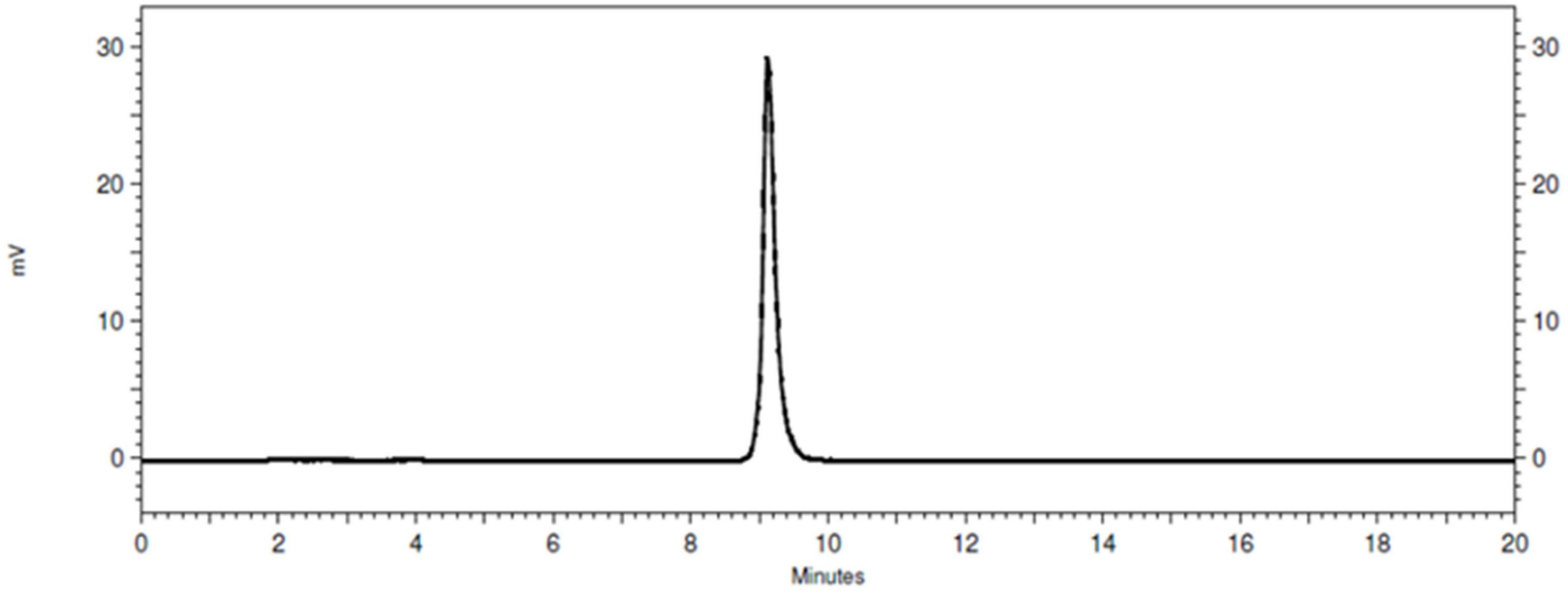
Representative radio-HPLC chromatogram of [^177^Lu]Lu-BQ7876. Determined purity for [^177^Lu]Lu-BQ7876 is >99%.

BQ7876 was successfully labeled with lutetium-177 with radiochemical yield exceeding 99%. The radio-HPLC chromatogram (**Figure 2**) demonstrated a single peak with the same retention time as the peak of BQ7876 loaded with stable lutetium. The results of radio-iTLC and radio-HPLC measurements of the radiochemical yield were in excellent agreement. Because of the high radiochemical purity, no further purification was required. PSMA-617 was successfully labeled with lutetium-177 with a radiochemical yield of 99.6%.

### 3.2 *In vitro* binding specificity

**Figure 3:**
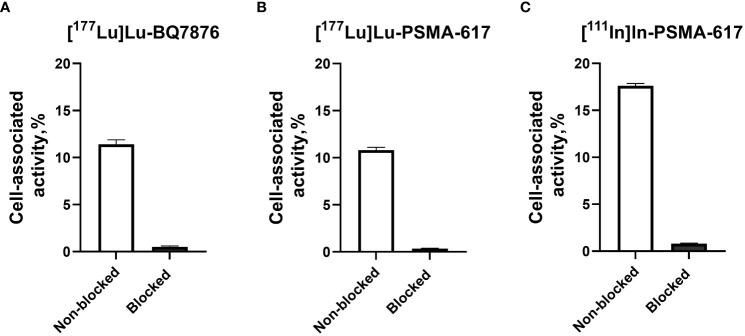
Specificity of [^177^Lu]Lu-BQ7876 **(A)**, [^177^Lu]Lu-PSMA-617 **(B)** and [^111^In]In-PSMA-617 **(C)** binding to PSMA-expressing PC-3pip prostate cancer cells *in vitro*. For blocking, a 100-fold molar excess of non-labeled BQ7876 or PSMA-617 was added before the addition of a radiolabeled counterpart. The final concentration of radiolabeled compounds was 1 nM. The data are presented as the average ± standard deviation of three samples.

The specificity of [^177^Lu]Lu-BQ7876, [^177^Lu]Lu-PSMA-617 and [^111^In]In-PSMA-617 binding to PSMA-expressing PC3-pip cells was studied by saturating the PSMA receptors with a large excess of unlabeled BQ7876 and PSMA-617, respectively. Saturation of PSMA with a large excess of unlabeled compound causes a highly significant (p< 5 x 10^-6^) reduction of binding of each labeled compound to cells, which demonstrates the saturable character of their binding (**Figure 3**).

### 3.3 Determination of half-maximal inhibitory concentration (IC_50_)

**Figure 4:**
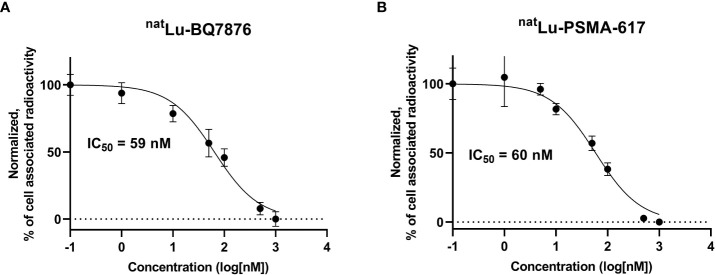
Inhibition of [^111^In]In-PSMA-617 binding to PC3-pip cells with ^nat^Lu-BQ7876 **(A)** and inhibition of ^177^Lu-PSMA-617 binding to PC3-pip cells with ^nat^Lu-PSMA-617 **(B)**. Data are presented as the mean value ± standard deviation (n=3).

The results of the comparison of inhibition of [^111^In]In-PSMA-617 binding to PSMA-expressing PC-3pip prostate cancer cells using ^nat^Lu-BQ7876 or ^nat^Lu-PSMA-617 are presented in **Figure 4**. The comparison suggested approximately equal inhibitory capacity of both compounds. The half-maximal inhibitory concentration for ^nat^Lu-BQ7876 was 59 nM (95% confidence interval from 44 to 80 nM), and the corresponding value for ^nat^Lu-PSMA-617 was 61 nM (95% confidence interval from 42 to 89 nM).

### 3.4 Binding affinity

**Figure 5:**
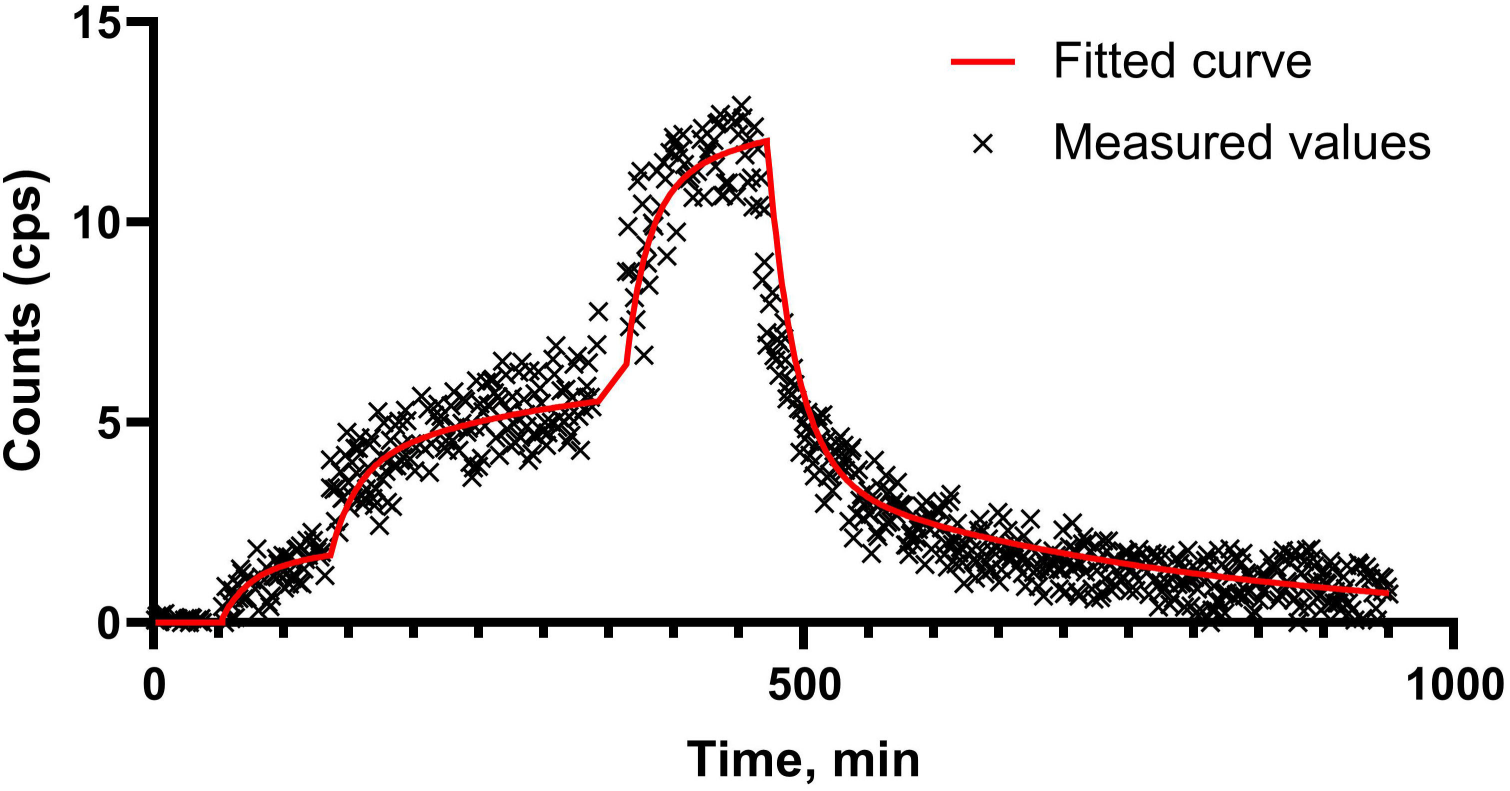
LigandTracer sensorgram of [^177^Lu]Lu-BQ7876 binding to PSMA-expressing PC3-pip cells.

The binding affinity of [^177^Lu]Lu-BQ7876 to PSMA was measured in real time at room temperature using PC3-pip cells. A typical sensorgram is presented in **Figure 5**. The binding of [^177^Lu]Lu-BQ7876 to living PC3-pip cells was characterized by rapid association, while the dissociation included a rapid and a slow phase. The measured rates of association (k_a_) and dissociation (k_d_) of radiolabeled peptide to PSMA positive PC3-pip cells were analyzed using one-to-two fitting model. The analysis indicated the presence of two interactions, one with affinity in the low nanomolar range (K*_D1_ =*3.8 nM, k_a_ = 1.4 ×10^4^ M^-1^s^-1^, k_d_ =5.7×10^-5^ s^-1^) and another one - approximately six times weaker (K*_D2_ =*25 nM, k_a_ = 3.2 ×10^4^ M^-1^s^-1^, k_d_ = 7.5×10^-4^ s^-1^).

### 3.5 Cellular processing and internalization

**Figure 6:**
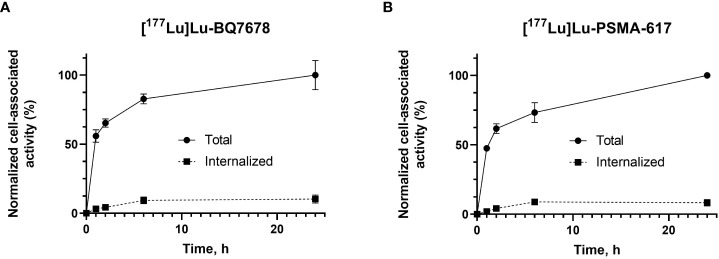
Cellular processing and internalization of [^177^Lu]Lu-BQ7876 **(A)** and [^177^Lu]Lu-PSMA-617 **(B)** by PSMA-expressing PC3-pip cells during continuous incubation over 24 h Data are presented as average ± standard deviation (n =3); error bars may not be visible when they are smaller than symbols.

The comparison of cellular processing of [^177^Lu]Lu-BQ7876 and [^177^Lu]Lu-PSMA-617 by PSMA-expressing PC-3pip cells is shown in **Figure 6**. The patterns of binding and processing of both substances were quite similar and were characterized by a rapid association. The internalization of both compounds was relatively slow: the internalized fraction of [^177^Lu]Lu-BQ7876 in PC3-pip cells was 8.0 ± 2.2% and the internalized fraction of PSMA-617 reached 10.0 ± 2.9% of the total cell-associated activity after 24 h of continuous incubation.

### 3.6 Biodistribution of [^177^Lu]Lu-BQ7876

**Figure 7:**
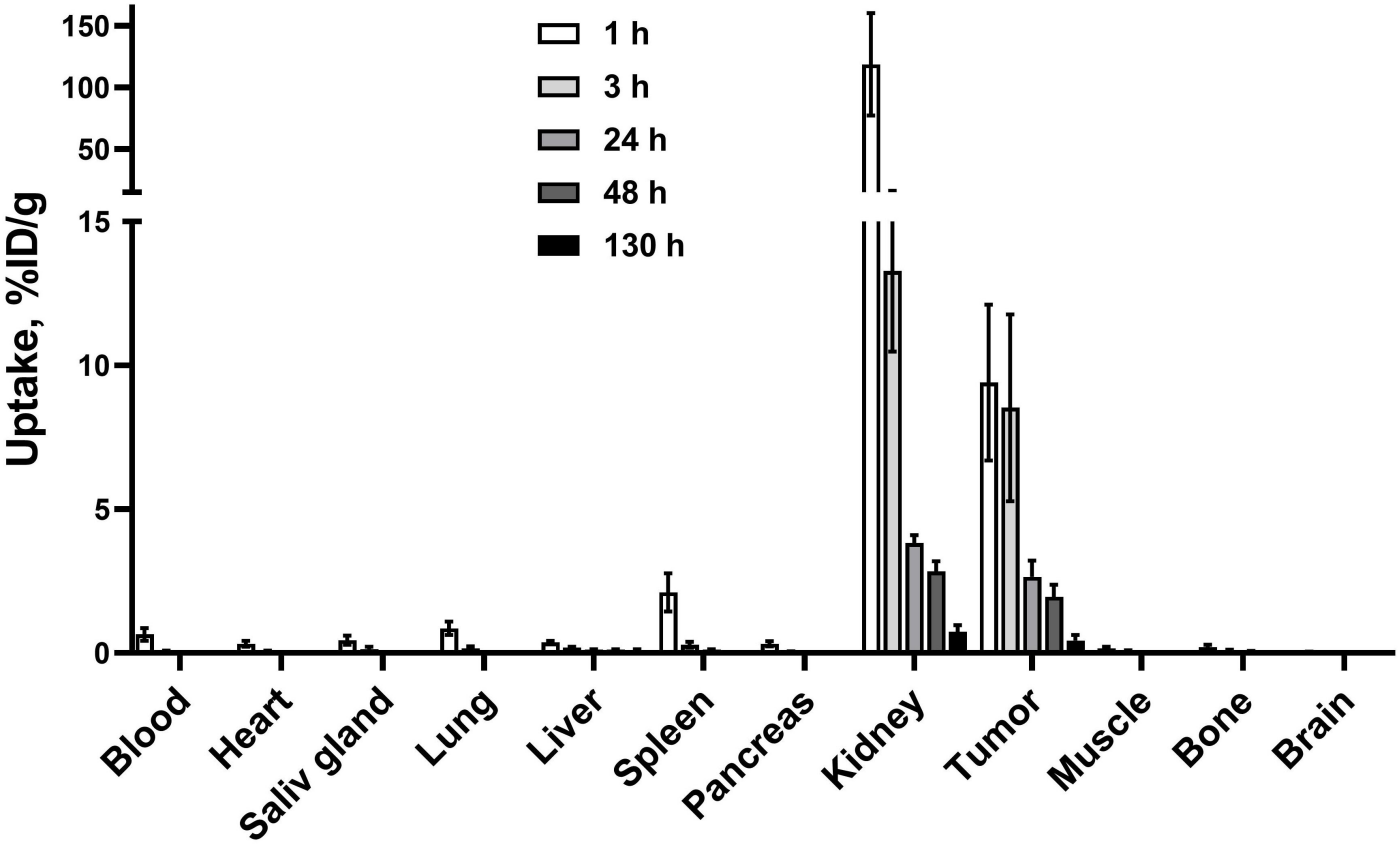
Biodistribution of [^177^Lu]Lu-BQ7876 in female BALB/C nu/nu mice bearing PC3-pip xenografts. Data are presented as the average value and standard deviation (n = 4).

The biodistribution of [^177^Lu]Lu-BQ7876 in Balb/c nu/nu mice bearing PC3-pip prostate cancer is presented in **Figure 7** and **Supplementary Table 1**. [^177^Lu]Lu-BQ7876 was cleared from the blood and normal tissues rapidly. Activity uptake decreased with time in all of studied organs and tissues. By 1 h after injection, the highest activity was found in the kidneys (118 ± 42%ID/g). Noticeable uptake was found also in the spleen (2.1± 0.7%ID/g). The uptake in other normal tissue was below 1%ID/g already at this time point. However, the elevated uptake in kidneys decreased rapidly, and by 3 h after injection, the renal uptake (13± 3%ID/g) did not differ significantly from tumor uptake (9± 3%ID/g). By 24 h after injection, the uptake was non-measurable in the majority of normal tissues. Tumor uptake was stable between 1 and 3 h followed by a slow decline.

### 3.7 *In vivo* binding specificity

**Figure 8:**
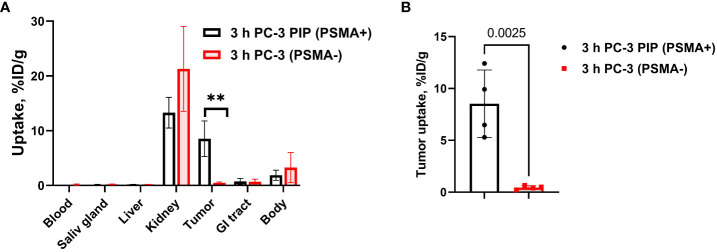
Specificity of PSMA targeting *in vivo*. **(A)** Comparison of general biodistribution of [^177^Lu]Lu-BQ7876 in mice bearing PSMA-positive PC3-pip and PSMA-negative PC3 xenografts. **(B)** Comparison of uptake in PC3-pip and PC3 xenografts. Data are presented as the mean ± SD (n = 4). P-value in unpaired t-test for xenografts is provided, **p < 0.005.

To confirm that the uptake of [^177^Lu]Lu-BQ7876 in PC3-pip xenografts was PSMA-dependent, the biodistribution of this compound was measured in mice bearing PSMA-negative PC3 xenografts (**Figure 8**; **Supplementary Table 1**). Expectedly, there was no significant difference between uptake of [^177^Lu]Lu-BQ7876 in normal organs and tissues (**Figure 8A**). However, the uptake in PSMA-positive PC3-pip xenografts was 19-fold higher than in PSMA-negative PC3 xenografts (p<0.005) (**Figure 8B**). This showed that tumor uptake of [^177^Lu]Lu-BQ7876 depends on the level of PSMA expression.

### 3.8 *In vivo* comparison with [^177^Lu]Lu-PSMA-617

**Figure 9:**
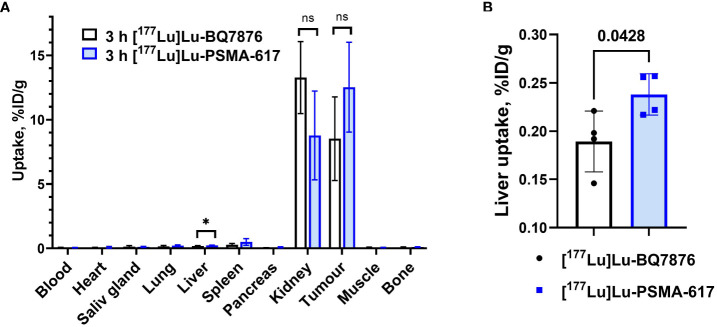
**(A)** Comparison of biodistribution of [^177^Lu]Lu-BQ7876 and [^177^Lu]Lu-PSMA-617 in female BALB/C nu/nu mice bearing PC3-pip xenografts at 3 h p.i. **(B)** Liver uptake of [^177^Lu]Lu-BQ7876 and [^177^Lu]Lu-PSMA-617. Data are presented as the average value and standard deviation (n =4). P-value in unpaired t-test for liver is provided, *p < 0.05; ns, nonsignificant.

Comparison between biodistribution of [^177^Lu]Lu-BQ7876 and [^177^Lu]Lu-PSMA-617 in female BALB/C nu/nu mice bearing PC3-pip xenografts at 3 h p.i is shown in **Figure 9** and **Supplementary Table 1**. The tumor uptake of both compounds did not differ significantly. There was no significant difference between the uptake of both compounds in almost all normal tissues (**Figure 9A**). However, the uptake of [^177^Lu]Lu-BQ7876 was 1.3-fold lower in the liver compared with the uptake of [^177^Lu]Lu-PSMA-617 (**Figure 9B**).

### 3.9 microSPECT/CT imaging

**Figure 10:**
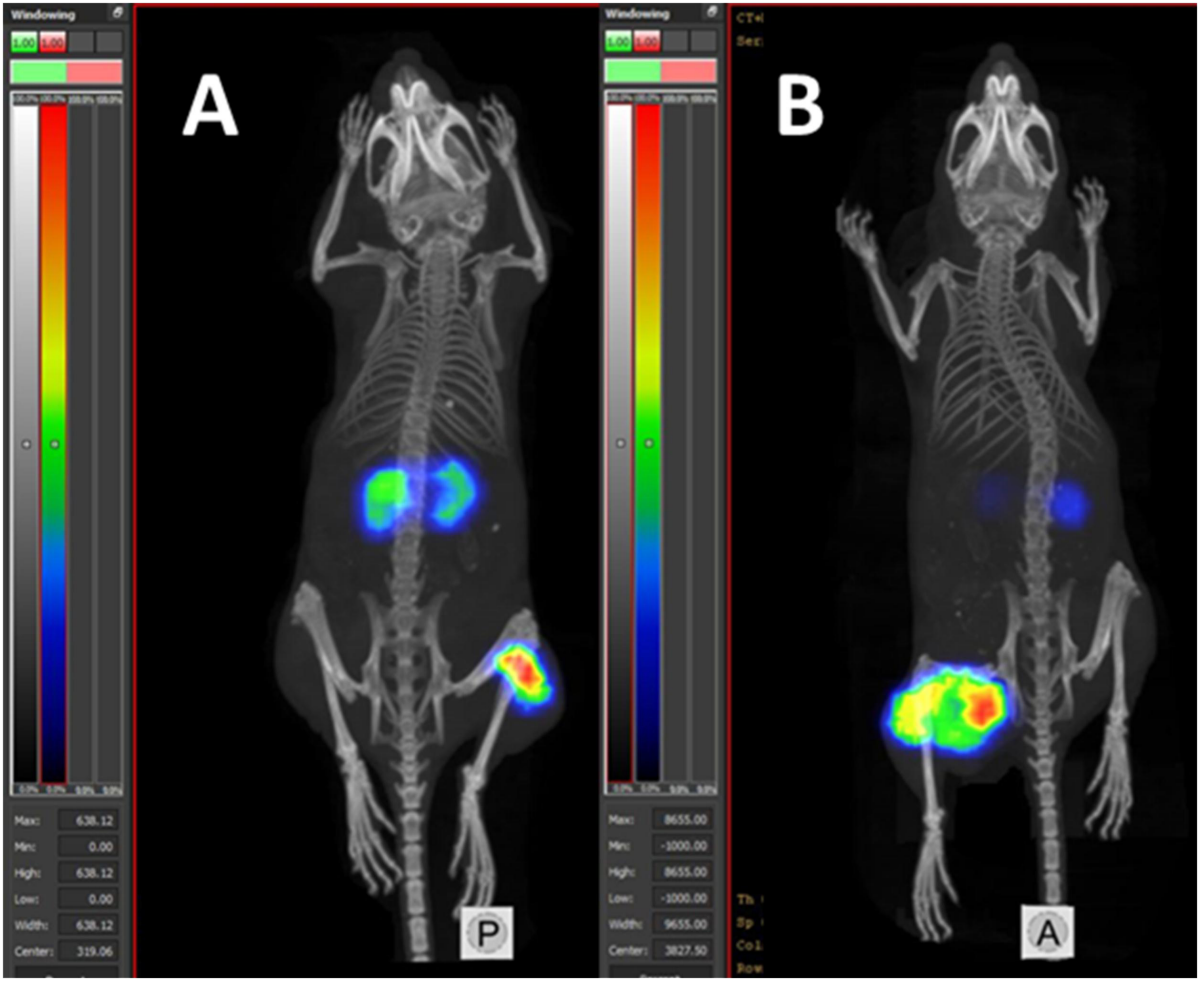
MicroSPECT/CT imaging of Balb/c nu/nu mice with PC3-pip PC xenografts using [^177^Lu]Lu-BQ7876 **(A)** and [^177^Lu]Lu-PSMA-617 **(B)** at 3 pi.

Results of microSPECT/CT imaging (**Figure 10**) were in agreement with the biodistribution data. Both [^177^Lu]Lu-BQ7876 and [^177^Lu]Lu-PSMA-617 accumulated avidly in tumors. The only other site of activity accumulation were the kidneys. No noticeable accumulation of activity in any other organ or tissue was detected.

### 3.10 Dosimetry estimation

Predicted absorbed doses in humans after injection of [^177^Lu]Lu-BQ7876, which were calculated using ex vivo data and software OLINDA/EXM 1.0, are provided in **Table 1**. The highest absorbed dose was expected in the kidneys, followed by the large intestines wall, gall bladder wall, heart wall, stomach wall and liver. The effective dose estimation was 0.00423 Sv/GBq (effective dose equivalent 0.00782 Sv/GBq).

**Table 1:** Estimation of absorbed doses (mGy/MBq) in humans after injection of [^177^Lu]Lu-BQ7876.

Organ	Absorbed dose	Organ	Absorbed dose
**Adrenals**	0.000468	**Lungs**	0.000526
**Brain**	0.0000435	**Muscle**	0.000125
**Breasts**	0.000185	**Pancreas**	0.000458
**Liver**	0.00121	**Red marrow**	0.000326
**Gallbladder wall**	0.000371	**Osteogenic cells**	0.00238
**Lower large intestine wall**	0.00938	**Skin**	0.000185
**Small intestine**	0.000927	**Spleen**	0.00179
**Stomach wall**	0.00128	**Testes**	0.00018
**Upper large intestine wall**	0.00595	**Thymus**	0.000203
**Heart wall**	0.00242	**Thyroid**	0.000179
**Kidneys**	0.106	**Prostate**	0.000239
**Urinary bladder wall**	0.000209	**Total body**	0.000813
**Effective dose (Sv/GBq)**	0.00423		
**Effective dose equivalent (Sv/GBq)**	0.00782		

The values are extrapolations from mouse data.

## 4 Discussion

PCa is one of the most common malignant tumors and when a metastatic PCa becomes hormone-refractory, there are only a few options left for treatment. Most metastatic PCa tumors have a high level of PSMA expression, which makes PSMA a perspective target for PCa treatment (27–29). Although [^177^Lu]Lu-PSMA-617 has demonstrated impressive results, the treatment has shown mainly partial responses in the Phase III VISION trial (20), and a search for more efficient targeting agents is well warranted.

In this project, BQ7876 demonstrated attractive features. Labeling of BQ7876 with lutetium-177 resulted in radiochemical purity over 99% (**Figure 2**), which makes its use for clinical trials possible without purification. Moreover, it opens the way for development of a simple two-vials kit for the labeling. Activity uptake decreased with time in all of studied organs and tissues including organs accumulating free lutetium (liver, spleen and bones (30);) that demonstrated stability of metal-DOTA complex in blood circulation. The *in vitro* binding of [^177^Lu]Lu-BQ7876 to PSMA-expressing PC3-pip cells was saturable, i.e. specific and approximately on the same level as the binding of [^177^Lu]Lu-PSMA-617, which is currently the gold standard in the development of PSMA-targeting therapeutic radiopharmaceuticals (**Figure 3**). Further *in vitro* studies showed that IC_50_ value for [^177^Lu]Lu-BQ7876 is close to the value for [^177^Lu]Lu-PSMA-617 (**Figure 4**), i.e. the [^177^Lu]Lu-BQ7876 binding strength to living PSMA-expressing PCa cells is as equally good as the binding strength of [^177^Lu]Lu-PSMA-617. This is encouraging because our previous experience demonstrated that modification of the linker part might change the affinity by three orders of magnitude (21). The binding of [^177^Lu]Lu-BQ7876 to PC3-pip cells was rapid (**Figures 5**, **6**), but LigandTracer sensorgrams showed an appreciable fraction of labeled pseudo-peptide with a rapid release. The analysis of the kinetics of [^177^Lu]Lu-BQ7876 binding to PC3-pip cells in conditions excluding internalization (room temperature) indicated the presence of two interactions, one with affinity in the low nanomolar range (K_D1_ = 3.8 nM) and another one with approximately a six times weaker affinity (K_D2_ = 25 nM). The weaker interaction was characterized by a noticeable more rapid dissociation rate (k_d_ = 7.5×10^-4^ s^-1^) than the stronger one (k_d_ = 5.7×10^-5^ s^-1^). It has been reported that PSMA is expressed on tumor cells as a noncovalent homodimer (31). It could be speculated that binding of a ligand to one of monomeric unit results in conformational changes of this unit, which influences the conformation of the second PSMA unit. It is conceivable that even a subtle change of conformation might result in different binding strength including the rate of the dissociation of bound ligand. It has to be noted that the affinity characterizes only a strength of binding of targeted agent to its molecular target in the equilibrium conditions. However, internalization of an agent with a residualizing label (such as ^177^Lu) makes its binding to malignant cells irreversible and only association rate plays a role. Therefore, we performed a direct, head-to-head, comparison of cellular processing of [^177^Lu]Lu-BQ7876 and [^177^Lu]Lu-PSMA-617. The result of such comparison shows that internalization of [^177^Lu]Lu-PSMA-617 and [^177^Lu]Lu-BQ7876 is equally slow (**Figure 6**). Thus, one could not expect big differences in binding of [^177^Lu]Lu-BQ7876 and [^177^Lu]Lu-PSMA-617 to cancer cells.

Evaluation of the biodistribution of [^177^Lu]Lu-BQ7876 in nude mice bearing PC3-pip xenografts demonstrated an efficient targeting of PSMA-expressing tumors (**Figures 7**, **10**), and 1 h after injection, the tumor uptake was higher than the uptake in any tissue excluding kidneys. Renal activity, however, decreased rapidly within the first hours after injection. The direct comparison demonstrated that uptake in PSMA-positive PC3-pip tumors was much higher than in PSMA-negative PC3 tumors (**Figure 8B**). Such difference clearly demonstrates that uptake of [^177^Lu]Lu-BQ7876 in PC3-pip xenografts is PSMA-mediated, i.e. true targeting takes place.

It is essential to compare the targeting properties of [^177^Lu]Lu-BQ7876 and [^177^Lu]Lu-PSMA-617. The majority of researchers use PSMA-transfected PC3-pip cells as a model for *in vitro* and *in vivo* studies on PSMA targeting. However, this cell line is quite an artificial system, and experts know that PSMA expression declines with time. Therefore, a comparison with literature data might be unreliable. For this reason, we performed the comparison head-to-head, in the same batch of mice with PC3-pip xenografts (**Figures 9**, **10**). The tumor uptake of [^177^Lu]Lu-BQ7876 and [^177^Lu]Lu-PSMA-617 did not differ significantly at 3 h after injection. The only significant difference was observed between the uptakes in the liver, where uptake of [^177^Lu]Lu-BQ7876 was significantly lower. This is a positive feature of [^177^Lu]Lu-BQ7876 because lower activity uptake in normal organs reduces the dose burden to patients. One could state, that [^177^Lu]Lu-BQ7876 is at least not inferior to [^177^Lu]Lu-PSMA-617 in animal studies. Apparently, animal models cannot reflect all aspects of the biodistribution of targeted therapeutics in humans, and a Phase I clinical trial is, therefore, necessary for the complete evaluation of [^177^Lu]Lu-BQ7876.

Estimation of absorbed doses in healthy organs and tissue in humans after administration of [^177^Lu]Lu-BQ7876 is provided in **Table 1**. The effective dose was calculated to be 0.00423 Sv/GBq, Obviously, upscaling from mice to humans is associated with multiple uncertainties (32). On the other hand, our upscaling of dosimetry of a bombesin-based imaging probe [^99m^Tc]Tc-maSSS-PEG2-RM26 (effective dose 0.00349 mSv/MBq) (33) showed to be in a reasonably good agreement with clinical data (0.0044 ± 0.0006 mSv/MBq) (34). As a first approximation, we compared the upscaling to humans of absorbed doses in different organs calculated for [^177^Lu]Lu-BQ7876 in this study and for [^177^Lu]Lu-PSMA-617 (35). Both calculation utilize the same formula for upscaling and the same software (OLINDA). The results of the comparisons (**Supplementary Table 2**) suggest that the injection of [^177^Lu]Lu-BQ7876 would result in somewhat lower absorbed doses to normal organs compared to [^177^Lu]Lu-PSMA-617. Still, both on-target and off-target interactions of targeting agents might be different in animal models and in humans. Strictly speaking, only clinical evaluation of distribution and dosimetry of [^177^Lu]Lu-BQ7876 (and comparison with an ample information available for [^177^Lu]Lu-PSMA-617) would show if [^177^Lu]Lu-BQ7876 offers any advantage in clinics.

The results of this study suggest that [^177^Lu]Lu-BQ7876 binds specifically to PSMA-expressing PCa cells *in vitro* and *in vivo*, and has an affinity to PSMA in a similar range as [^177^Lu]Lu-PSMA-617. Biodistribution studies in mice demonstrated that targeting properties of [^177^Lu]Lu-BQ7876 are not inferior to the properties of [^177^Lu]Lu-PSMA-617.

## Data availability statement

The original contributions presented in the study are included in the article/**Supplementary Material**. Further inquiries can be directed to the corresponding author.

## Ethics statement

The animal study was approved by Local Ethics Committee for Animal Research (Uppsala, Sweden). The study was conducted in accordance with the local legislation and institutional requirements.

## Author contributions

VT, UR, and AO contributed to conception and design of the study. FL performed peptide synthesis. AA and KS performed radiolabeling. KS and VB performed *in vitro* studies. AA, KS, VB, MO and AO performed *in vivo* studies. AO performed imaging studies. VT performed dosimetry estimation. VT wrote the first draft of the manuscript. KS wrote sections of the manuscript. AO, MO, FL, and UR performed editing of the first draft. All authors contributed to the article and approved the submitted version.
