# Supplementary material for: 177Lu-labeled PSMA targeting therapeutic with optimized linker for treatment of disseminated prostate cancer; evaluation of biodistribution and dosimetry
Source: Front Oncol. 2023 Sep 27;13:1221103. doi: 10.3389/fonc.2023.1221103 (PMC10565663; doi:10.3389/fonc.2023.1221103)

Supplementary Material

^177^Lu-labelled PSMA targeting therapeutic with optimized linker for treatment of disseminated prostate cancer; evaluation of biodistribution and dosimetry.

Ayman Abouzayed, Kamila Seitova, Fanny Lundmark, Vitalina Bodenko, Maryam Oroujeni, Vladimir Tolmachev, Ulrika Rosenström, Anna Orlova

*** Correspondence:** Anna Orlova, anna.orlova@ilk.uu.se

Table S1: Biodistribution results of [^177^Lu]Lu-BQ7876 and [^177^Lu]Lu-PSMA-617 in Balb/c nu/nu mice bearing PC3-pip tumors, expressed as average %IA/g ± SD, n = 4. Data in parentheses for 3 h pi of [^177^Lu]Lu-BQ7876 are for the group bearing PSMA-negative PC3 xenografts.

|  | **BQ7876** | | | | | **PSMA-617** |
| --- | --- | --- | --- | --- | --- | --- |
|  | 1 h | 3 h | 24 h | 48 h | 130 h | 3 h |
| Blood | 0.7±0.2 | 0.07±0.02 (*0.1±0.1*) | 0.01±0.00 | ** | *** | 0.06±0.02 |
| Heart | 0.3±0.1 | 0.06±0.03 | 0.02±0.00 | ** | *** | 0.08±0.07 |
| Salivary gland | 0.5±0.2 | 0.13±0.09 (*0.19±0.07*) | 0.02±0.01 | ** | *** | 0.12±0.04 |
| Lung | 0.9±0.2 | 0.16±0.07 | 0.03±0.01 | ** | *** | 0.21±0.07 |
| Liver | 0.37±0.05 | 0.19±0.03 (*0.18±0.04*) | 0.12±0.01 | 0.12±0.01 | 0.08±0.05 | 0.24±0.02* |
| Spleen | 2.1±0.7 | 0.3±0.1 | 0.12±0.01 | ** | *** | 0.5±0.3 |
| Pancreas | 0.32±0.09 | 0.05±0.01 | 0.02±0.01 | ** | *** | 0.09±0.04 |
| Kidney | 119±42 | 13±3 (*21±8*) | 3.8±0.3 | 2.8±0.4 | 0.7±0.2 | 9±3 |
| Tumor | 9±3 | 9±3 (*0.5±0.2**) | 2.7±0.6 | 2.0±0.4 | 0.4±0.2 | 12±3 |
| Muscle | 0.16±0.06 | 0.05±0.05 | 0.01±0.00 | ** | *** | 0.07±0.02 |
| Bone | 0.21±0.08 | 0.09±0.03 | 0.06±0.01 | ** | *** | 0.11±0.02 |
| Brain | 0.04±0.02 | 0.01±0.00 | ** | ** | *** | *** |

* indicates a significant difference between corresponding groups;

** activity content was below measurable limits;

*** samples were not collected.

Table S2. Dosimetry comparison. Estimated absorbed doses (mGy/MBq) in humans after injection of [^177^Lu]Lu-BQ7876 and [^177^Lu]Lu-PSMA-617. The values are extrapolated from data concerning biodistribution in mice.

| Organ | Absorbed dose (mGy/MBq) | |
| --- | --- | --- |
|  | [^177^Lu]Lu-BQ7876,  This work | [^177^Lu]Lu-PSMA-617, Data from Kuo et al. 2018* |
| Adrenals | 4.68E-04 | 7.64E-03 |
| Brain | 4.35E-05 | 4.32E-04 |
| Gallbladder wall | 3.71E-04 | 8.76E-04 |
| Small Intestine | 9.27E-04 | 1.72E-03 |
| Stomach wall | 1.28E-03 | 1.69E-03 |
| Heart wall | 2.42E-03 | 1.00E-03 |
| Kidneys | 1.06E-01 | 1.11E-01 |
| Liver | 1.21E-03 | 1.37E-03 |
| Lungs | 5.26E-04 | 1.81E-03 |
| Pancreas | 4.58E-04 | 7.60E-04 |
| Red marrow | 3.26E-04 | 7.59E-04 |
| Osteogenic cells | 2.38E-03 | 8.30E-04 |
| Spleen | 1.79E-03 | 4.42E-03 |
| Testes | 1.80E-04 | 1.05E-03 |
| Thymus | 2.03E-04 | 7.19E-04 |
| Thyroid | 1.79E-04 | 7.11E-04 |
| Prostate | 2.39E-04 | 1.17E-03 |
| Total body | 8.13E-04 | 1.70E-03 |

Kuo HT, Merkens H, Zhang Z, Uribe CF, Lau J, Zhang C, Colpo N, Lin KS, Bénard F. Enhancing Treatment Efficacy of 177Lu-PSMA-617 with the Conjugation of an Albumin-Binding Motif: Preclinical Dosimetry and Endoradiotherapy Studies. Mol Pharm. 2018 Nov 5;15(11):5183-5191.

Figure S1. BQ7876: Analytical high-performance liquid chromatography (HPLC) performed on a Dionex UltiMate 3000 HPLC system with a Bruker amazon SL ion trap mass spectrometer using a Penomenex Kinetex C18 column (50 × 3.0 mm, 2.6 µm particle size, 100 Å pore size) with gradients of H2O/CH3CN/0.05%HCOOH as mobile phase at a flow rate of 1.5 mL/min.


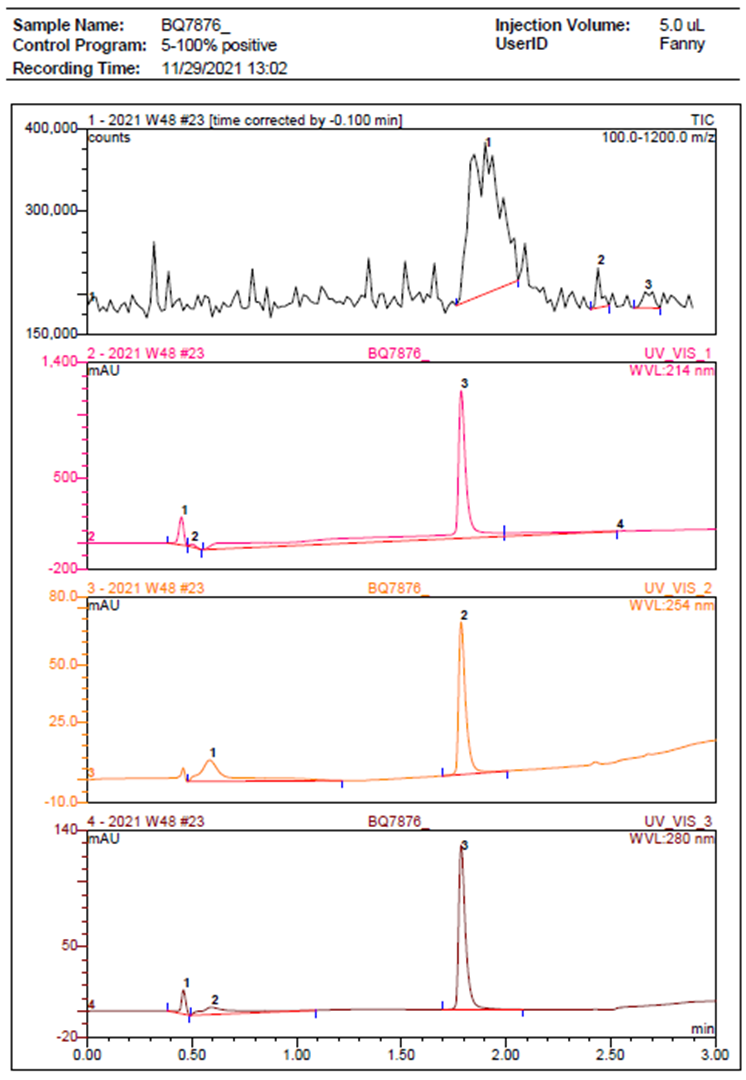


Figure S2. BQ7876: Electrospray ionization (ESI) MS using a Penomenex Kinetex C18 column (50 × 3.0 mm, 2.6 µm particle size, 100 Å pore size) with gradients of H2O/CH3CN/0.05%HCOOH as mobile phase at a flow rate of 1.5 mL/min.


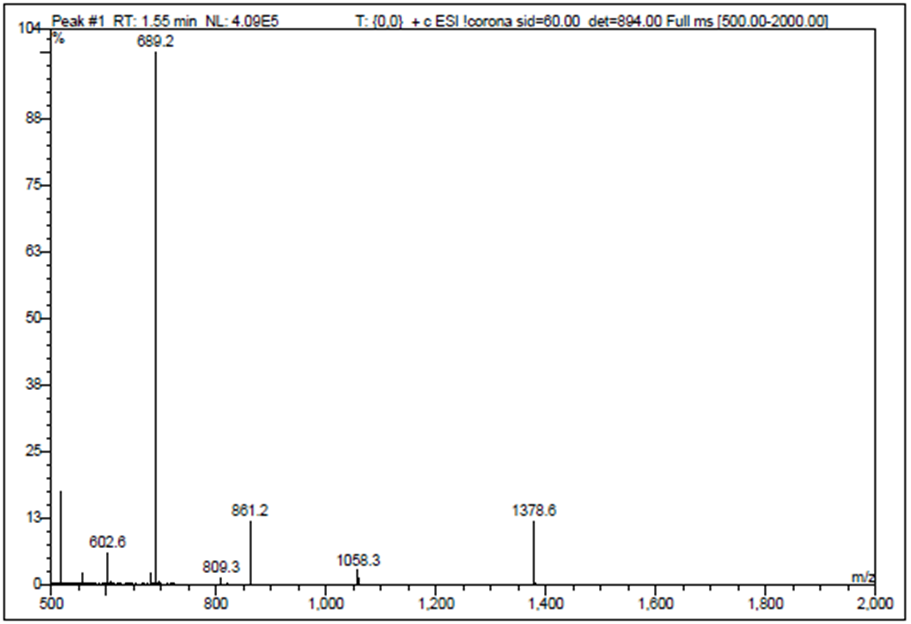

Supplement: Supplementary file 1 [file DataSheet_1.docx]
